# Esketamine-based opioid-free anaesthesia alleviates postoperative nausea and vomiting in patients who underwent laparoscopic surgery: study protocol for a randomized, double-blinded, multicentre trial

**DOI:** 10.1186/s13063-022-07003-3

**Published:** 2023-01-06

**Authors:** Hai-yan Chen, Xiao-yan Meng, Hao Gao, Hui Liu, Hai-Bo Qiu, Jun Lu, Jin-Chao Song

**Affiliations:** 1 Department of Anaesthesiology, Shidong Hospital of Shanghai, University of Shanghai for Science and Technology, Shiguang Rd., No. 999, Shanghai, China; 2 Department of Anaesthesiology, Eastern Hepatobiliary Surgery Hospital, Second Military Medical University, Changhai Rd., No.225, Shanghai, China; 3 Department of Anaesthesiology, Shuguang Hospital, Shanghai University of Traditional Chinese Medicine, Shanghai, China; 4 Department of Anaesthesiology, Shanghai East Hospital, Tongji University School of Medicine, Jimo Rd., No.150, Shanghai, China

**Keywords:** Esketamine, Opioid-free anaesthesia, Postoperative nausea and vomiting

## Abstract

**Background:**

Although opioids are commonly prescribed in clinical anaesthesia, the significant side effects attributed to their overuse are raising increasing concerns. One way to reduce perioperative opioid consumption is to apply opioid-reduced anaesthesia (ORA) and even opioid-free anaesthesia (OFA), which involves regional techniques, neuraxial anaesthesia, nonopioid analgesics or combined use. The aim of this study was to investigate whether the application of OFA by using esketamine in intraoperative analgesia could minimize the side effects of postoperative nausea and vomiting (PONV), as well as other short-term side effects related to anaesthesia.

**Methods/design:**

The study was designed as a prospective, randomized, controlled, multicentre trial. A total of 278 patients were enrolled; participants were nonsmoking female patients aged 18–50 years and scheduled for laparoscopic appendectomy or cholecystectomy, ASA at I–III, with no serious physical or mental diseases. Both groups received usual perioperative care except for the analgesic medication of either esketamine or sufentanil. The primary outcome was the incidence of PONV 3 days after surgery. Secondary outcomes included recovery status, pain, sedation level and overall recovery, delirium and cognition, anxiety and depression and total consumption of analgesic agents.

**Discussion:**

This trial may show that the synergy of esketamine and propofol anaesthesia reduces PONV as well as other short-term adverse events, thereby providing a better safety and satisfaction profile of ERAS for laparoscopic appendectomy and cholecystectomy.

**Trial registration:**

Chinese Clinical Trial Registry ChiCTR2100047169. Registered on June 9, 2021

## Introduction

Opioids are commonly prescribed in the peri-operative period for analgesia and pain management. However, immediate adverse reactions or the long-term sequelae of chronic effects attributed to the overuse of opioids perioperatively may lead to significant morbidity and mortality [1]. It seems that the incidence of opioid-related adverse events is generally underestimated in the clinic, as previous research has reported that opioid-related adverse events may occur in 12% of surgeries [2]. One common side effect of opioids is postoperative nausea and vomiting (PONV), which contributes to a delay in oral intake and mobilization with a prolonged hospital length of stay or unexpected hospital readmissions [3]. Other side effects including pruritis, respiratory depression, and constipation could also lead to prolonged hospital admissions. In addition, recent evidence also reveals the potential role of opioids in cancer recurrence [4, 5]. Despite all these severe adverse events, the intraoperative use of opioids remains a central part of anaesthesia practice.

As doubts have increased, more attention has been given to reducing perioperative opioid consumption. The recently developed enhanced recovery after surgery (ERAS) protocols have promoted several opioid alternatives, including neuraxial anaesthesia, peripheral nerve blocks and nonopioid adjuncts. Opioid-free anaesthesia (OFA) is an opioid-sparing technique, which focuses on multimodal or balanced analgesia [6, 7]. Generally, the implementation of opioid-reduced anaesthesia (ORA) or OFA often involves regional techniques, neuraxial anaesthesia, nonopioid analgesics or their combined use. Among them, nonopioid medications have received wide attention, including acetaminophen, nonsteroidal anti-inflammatory drugs, alpha-2 agonists, *N*-methyl-d-aspartate (NMDA) receptor antagonists, gabapentioids and antidepressants [8, 9]. Substantial evidence has suggested that using OFA with nonopioid adjuncts not only minimizes the adverse effects of opioids but also enables earlier ambulation and return of bowel function and ultimately facilitates enhanced recovery after surgery (ERAS) protocols [10, 11].

Esketamine, a right-handed split of ketamine, might be a potential substitute for opioids in ORA or OFA. Esketamine is a noncompetitive *N*-methyl-d-aspartate (NMDA)-receptor antagonist, and its anaesthetic effect is approximately twofold higher than that of racemic ketamine, with a lower incidence of psychotropic side effects at equivalent doses [12, 13]. In addition, due to a ketamine-induced increase in sympathetic tone, esketamine results in less respiratory depression and hypotension than other anaesthetics and analgesics [14]. Studies also report that esketamine creates less impairment in concentration capacity and primary memory and fewer cardiopulmonary adverse effects and may lead to a faster recovery [15, 16]. Furthermore, one recent study suggested that esketamine could be an attractive additive to propofol sedation instead of opioids, as an adequate level of sedation and analgesia could be achieved with less propofol [17].

## Methods/design

### Aim of the study

We hypothesize that the application of OFA by using esketamine in intraoperative analgesia may minimize the side effects of postoperative nausea and vomiting (PONV), as well as other short-term side effects related to anaesthesia, while maintaining the same satisfaction level of patients. To test this hypothesis, we compared the two groups that underwent laparoscopic appendectomy and cholecystectomy: group E received propofol/esketamine sedation, and group F received propofol/sufentanil sedation. Both groups received standard deep sedation with propofol target-controlled infusion (TCI) provided by specialized sedation anaesthesia nurses. The primary objective of this study is to test the hypothesis that esketamine-based opioid-free anaesthesia alleviates postoperative nausea and vomiting in patients who underwent laparoscopic surgery.

### Study settings

The study is designed as a prospective, randomized, controlled, multicentre trial. The protocol is in accordance with the Standard Protocol Items: Recommendations for Interventional Trials (SPIRIT) statement [18]. This trial is registered at ClinicalTrial.gov with ID number ChiCTR2100047169. The sponsor of this trial is the Department of Anaesthesiology of the Shidong Hospital, University of Shanghai for Science and Technology. The sponsor is responsible for the collection, management, analysis and interpretation of the data as well as the writing of the report and the decision to submit the report for publication. The study is supported by the Yangpu District Good-Doctor Program.

The participating clinical centres are as follows: (1) Department of Anaesthesiology, Shidong Hospital of Shanghai, University of Shanghai for Science and Technology; (2) Department of Anaesthesiology, Eastern Hepatobiliary Surgery Hospital, Second Military Medical University; (3) Department of Anaesthesiology, Shuguang Hospital, Shanghai University of Traditional Chinese Medicine; and (4) Department of Anaesthesiology, Shanghai East Hospital, Tongji University School of Medicine.

### Inclusion and exclusion criteria

The inclusion criteria were as follows: (1) nonsmoking female patients who were 18–50 years old with a planned laparoscopic appendectomy or cholecystectomy from October 1, 2021, to October 1, 2022; (2) American Society of Anaesthesiologists (ASA) classification I–III; (3) no analgesic/sedative medication was used within 24 h before surgery; (4) no serious endocrine, cardiovascular and respiratory diseases before surgery; (5) no mental abnormality; and (6) provision of signed informed consent.

The exclusion criteria included patients who (1) had an allergic history of anaesthetic drugs or contraindications of esketamine, nonsteroidal anti-inflammatory drugs or dexmedetomidine; (2) had mental disorders, preoperatively screened using a neuropsychiatric inventory; (3) had poorly controlled or untreated hypertension with resting arterial pressure over 180/100 mmHg; (3) had addiction or dependence on opioid or hypnotic drugs; and (4) had respiratory infection or asthma before surgery.

Patients who were enrolled but met one of the following criteria were excluded from the study: (1) incomplete case report form or lost to follow-up and failed to make an effective and safety assessment, (2) change of anaesthesia method due to unexpected incident or surgery requirement during the surgery, (3) operation could not be continued due to unexpected incident during the surgery and (4) other diseases post-surgery required urgent treatment.

### Dropout criteria

The dropout criteria are as follows: (1) withdrawal of consent by the participants or their legal representative and (2) loss to follow-up.

### Ethics issues

This study was approved by the Ethics Committee of Shidong Hospital, University of Shanghai for Science and Technology (Ethics number: YPSDKY2021-02-009). Designated doctors explained this trial to interested potential participants in detail and provided them with the informed consent form. Participants were given at least 6 h to decide whether they wished to participate in the trial. The informed consent form was signed by the participant or his or her trustee or guardian, and consent could be withdrawn at any time during the trial. Written informed consent and the patient’s baseline data were obtained before randomization. Moreover, participants were encouraged to contact the research team if they had any health concerns during the trial.

**Fig. 1:**
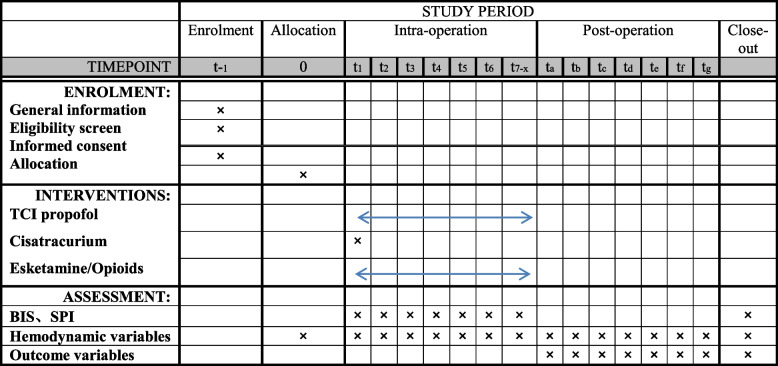
Schedule of enrolment, intervention and assessment according to the Standard Protocol Items: Recommendations for Interventional Trials (SPIRIT) statement. Time points for implement of the study: *t*_−1_: when enrolled, *t*_0_: allocation, *t*_1_: before anaesthesia, *t*_2_: TCI propofol reached equilibrium, *t*_3_: in 3 min after sufentanil or esketamine infusion, *t*_4_: immediately after intubation, *t*_5_: in 5 min after intubation, *t*_6_: pneumoperitoneum, *t*_7−*x*_ in each 15 min till the end of the surgery; *t*_*a*−*g*_ refers to 0, 2, 4, 8, 24, 48 and 72 h after anaesthesia

The schedule of enrolment, intervention and assessment is reported according to the SPIRIT statement (Fig. 1).

### Sequence generation and allocation concealment

Blocked randomization was conducted. A randomization sequence was generated using a computerized random number generator, and patients who met the enrolment criteria were randomized in a 1:1 ratio to either the control or esketamine anaesthesia group. Once the patient was qualified for the study, the project coordinator opened a sealed opaque envelope with random serial numbers. Only the statistician and the project coordinator knew the treatment allocation of each participant.

### Blinding

The study was performed as a double-blinded study. The esketamine (1 mg/kg) or sufentanil (0.5 μg/kg) used for induction of anaesthesia was diluted to 20 ml solution with normal saline, and another same dosage was also diluted to 20 ml solution, preparing for use during surgery if necessary. The drugs of the two groups were prepared by the nurse, who subsequently withdrew from the study and did not participate in anaesthesia management, postoperative follow-up and other affairs. Therefore, the anaesthesiologists were blinded to the grouping. All patients were blinded as well.

### Intervention

All patients fasted at least 6 h before surgery. Peripheral venous access was opened after entering the operating room, and antibiotic prophylaxis was given according to hospital standards. Measurements of heart rate (HR), electrocardiogram (ECG), noninvasive blood pressure (NIBP), respiratory rate (RR), oxygen saturation (SpO_2_), end-tidal carbon dioxide (etCO_2_), sedation level measured by BIS and nociception level by Surgical Pleth Index (SPI) were recorded.

Procedural sedation was performed by experienced anaesthesiologists. Both groups were sedated by a propofol TCI system (Propofol 1% MCT Fresenius). We started propofol TCI in both groups with a targeted plasma level of 2–4 μg/ml. Upon reaching this plasma level, 0.1–0.2 mg/kg cisatracurium besilate was administered. Group E was treated with 1 mg/kg esketamine (Ketanest S, Pfizer), and the infusion lasted for 30 s. Group F was treated with 0.5 μg/kg sufentanil (Rapifen, Janssen-Cilag), with infusion lasting for 30 s. The esketamine (1 mg/kg) or sufentanil (0.5 μg/kg) used for induction of anaesthesia was diluted to 20 ml solution with normal saline, and another same dosage was also diluted to 20 ml, preparing for use during surgery if necessary. One of three of the induction dosage (6.5 ml solution) was given each time if necessary. Under special circumstances, the nurse who had prepared the medicines provided the corresponding medicines again. Mechanical ventilation was applied followed by tracheal intubation; BIS was maintained at 40–60, ETCO_2_ was maintained at 35–45 mmHg and muscle relaxant was given as needed.

All anaesthetics were stopped when pneumoperitoneum stopped; then, muscle relaxant antagonists were applied according to their conditions, and endotracheal intubation was removed according to the anaesthesiologist’s judgement. “Ready for discharge” was declared when a Steward recovery score was ≥ 9. Parecoxib or flurbiprofen was applied intravenously when postoperative analgesia was necessary, and if parecoxib or flurbiprofen did not work well, peripheral nerve blocking was given as needed. Postoperative antiemetic drugs of dexamethasone, metoclopramide or 5-hydroxytryptamine antagonists for PONV were also applied accordingly as needed. 5-Hydroxytryptamine antagonists (4 mg ondansetron) for PONV were administered intravenously.

### Primary outcome

The primary outcome was the incidence of PONV 3 days after surgery. PONV is defined as a spectrum incorporating some combination of nausea, vomiting and/or retching in the postoperative period. Nausea and vomiting scales are also used to grade PONV severity [19]. The dosage and frequency of use of postoperative antiemetic drugs (metoclopramide or 5-hydroxytryptamine antagonist) were also recorded for analysis.

### Secondary outcomes

Variables indicating recovery status included the time to spontaneous respiration, awakening, eye opening and extubation after stopping anaesthetic agents. The scales used for patient pain, sedation level and overall recovery assessment included the visual analogue score (VAS), Modified Observer’s Assessment of Alertness/Sedation (MOAA/S), Bruggrmann Comfort Scale (BCS) [20] and 15-item Quality of Recovery Questionnaire (QoR-15) [21]. Delirium and cognition were assessed with the Confusion Assessment Method and Mini-Mental State Examination [22, 23]. Anxiety and depression were evaluated with the Hospital Anxiety and Depression Scale [24]. All scales were assessed at 0, 2, 4, 8, 24 and 72 h after waking. The total amount of propofol consumption, all drugs used during anaesthesia and analgesics for postoperative pain management were recorded. Side effects or adverse events were recorded for 3 postoperative days, and thereafter, the hospital stay time was recorded. The intraoperative variables collected included anaesthesia and surgery time, intraoperative medication, blood loss, fluid and blood transfusion, urine output, mean arterial pressure (MAP) and heart rate, time and duration of low blood pressure (MAP < 60 mmHg) and nociceptive level of SPI. The average percent change to baseline in mean arterial pressure and heart rate was also calculated. Percent change for arterial pressure = (MBPT1−*x* − MBPT0)/MBPT0 × 100; percent change for HR = (HRT1−*x* − HRT0)/HRT0 × 100. The recorded time points are as follows: after entering the operation room, patients were allowed to be quiet for 10 min, and blood pressure and heart rate were measured twice (15 min apart) and the average values were recorded as the basic blood pressure and heart rate (T0), when TCI propofol reached equilibrium (T1), 3 min after sufentanil or esketamine infusion (T2), immediately after intubation (T3), 5 min after intubation (T4), time to pneumoperitoneum (T5) and then every 15 min (T6−*x*) until the end of the surgery.

### Safety management and adverse events

An adverse event refers to an untoward medical occurrence that happens during the trial. Adverse events include but are not limited to PONV, mental disorders, respiratory depression or cardiocirculatory instability. All adverse events were treated immediately. All adverse events were documented and discussed in data monitoring committee (DMC) meetings.

### Data collection and management

The collected data including sociodemographic characteristics, peri-operative variables and various questionnaires are as mentioned above. To improve data quality, we set up a data monitoring committee consisting of a medical doctor, a statistician and a nurse. All survey data were checked for logical or clerical errors, with significant errors will be verified by the participant. Final survey data completed by all participants will be saved in a secure computer only used for saving data.

### Data monitoring

The data monitoring committee (DMC), composed of statisticians, principal investigators from each centre and representatives from the ethics committee, will be responsible for data monitoring. Members of the data monitoring committee are independent of the sponsors. The written report on trial progress from each centre will be submitted to the committee quarterly. Cases of adverse events and unexpected scenarios will be discussed at committee meetings.

### Sample size calculation

The sample size was calculated with PASS 15.0 (Number Cruncher Statistical Software, LLC, Kaysville, UT, USA). The hypothesis of this trial is that esketamine could be associated with fewer side effects of PONV than opioid anaesthesia. With *α* = 0.05 and a power of 90%, we assumed that esketamine anaesthesia may reduce the PONV rate from 37.3 to 20% according to a study by Ziemann-Gimmel et al. [25]; thus, a sample size of 139 for each group would be needed. According to previous studies, the general incidence of vomiting after surgical anaesthesia is approximately 30%, while the incidence of nausea is approximately 50% [26–28]. Therefore, proportions of 37.3% and 20% of PONV of patients in the F group and E group, respectively, were used to calculate the sample size.

### Statistical analysis

Statistical analyses will be performed using SPSS 25.0 (IBM SPSS Statistics, Armonk, NY). An intention-to-treat (ITT) approach will be used to analyse the data. All data will be checked for normal distribution using the Kolmogorov test. For normally distributed data, continuous variables will be analysed using the independent Student’s *t* test, and the variables will be presented as the mean ± standard deviation (SD). Nonnormally distributed data will be compared using the Mann‒Whitney *U* test where appropriate, and data will be presented as the median and interquartile range (IQR). For categorical variables, cross-tabulation and the Pearson chi-squared test will be applied, and variables will be categorized as numbers and/or percentages of the total. A logistic regression model will be applied to detect any potential confounding factors. To compare the continuous measurements of HR and NIBP between the groups, the area under the curve (AUC) for each value will be calculated over the different measurement time points during the procedure. To compare the continuous measurements of HR and NIBP between the groups, the area under the curve (AUC) for each value will be calculated over the different measurement time points during the procedure. Missing data are neglected or multiple imputation will be used when > 40% and identified as missing, not at random. A *p* value < 0.05 with two tails will be considered statistically significant.

## Discussion

In recent years, the perioperative use of opioids has caused an opioid crisis and has become a major public health issue worldwide. Opioids have a multitude of acute side effects including pruritis, respiratory depression, nausea and vomiting postoperatively. Among them, PONV has already been proven to be associated with prolonged hospital length of stay, unexpected hospital readmissions, increased medical expenses and patient dissatisfaction with the perioperative experience [29, 30]. Thus, one of the key elements of the implementation of ERAS protocols is to help patients minimize opioid use, without negatively impacting perioperative pain management or recovery [31].

OFA is essentially the practice of intraoperative anaesthesia without the use of intraoperative opioids and has been proven to be a feasible technique for safe sedation. One strategy for OFA is to increase nonopioid adjuncts during the implementation of multimodal analgesia or balanced analgesia. However, a substitute agent that can fully replace opioids perioperatively has not been found, and whether OFA is beneficial and can improve short-term and long-term patient outcomes remains unknown.

Recently, esketamine has received wide attention for its potential implications in treatment-resistant depression. In addition to its antidepressant effect, esketamine could also be an effective anaesthetic and analgesic agent used for surgical anaesthesia.

According to a recent study on Chinese patients, the administration of esketamine as a relatively small dose was generally safe for anaesthesia without consideration of sex differences [32]. In fact, several advantages of esketamine in clinical anaesthesia have recently been proven. First, it creates less impairment on patients’ spontaneous breathing and airway reflexes as well as circulation stability due to an increase in sympathetic tone. In addition, Eberl et al. [17] reported that low-dose esketamine reduces the total amount of propofol necessary for sedation during ERCP while proving satisfactory sedative effects. Furthermore, many studies report that a combination of propofol with ketamine may result in a better quality of sedation and analgesia, with shorter recovery time, better satisfaction of patients and fewer respiratory or cardiovascular side effects [33]. Despite all these benefits, one widespread concern of esketamine is that it can produce psychotomimetic effects that may be associated with cognitive impairment [13].

In this trial, we hypothesized that OFA with esketamine could reduce the PONV rate after laparoscopic surgery based on several possible underlying mechanisms. On the one hand, it certainly reduced the consumption of opioids perioperatively, which has been proven to be one of the main risk factors for PONV [34]. On the other hand, esketamine is also known for its effective effects in maintaining spontaneous breathing and airway reflexes during anaesthesia; in addition, it can increase sympathetic tone and result in less hypotension and cardiac depression [35]. The reduced interference of esketamine on respiratory and circulatory systems during surgery also provides better conditions for ERAS in patients.

However, research on the advantages or disadvantages of esketamine in surgical anaesthesia remains deficient, and further randomized trials with larger sample sizes are urgently needed in this area. In this study, we also plan to investigate intraoperative circulatory stability as well as other recovery indices for esketamine in anaesthesia for laparoscopic appendectomy and cholecystectomy.

A limitation of our study could be that no patients in either group accepted pretreatment for PONV, in case it may cover the potential anti-PONV effect of esketamine. As a rescue regimen, we allowed the prescription of antivomiting agents postoperatively as needed, and the consumption of postoperative antivomiting agents was also recorded and analysed. Additionally, we defined side effects and cognitive dysfunctions according to the SIVA consensus statement for standardized definitions and terminology for sedation-related adverse events [36]. However, their clinical impact cannot be determined when a sedation specialist provides adequate rescue manoeuvres during such events.

In summary, the aim of our trial is to demonstrate that the synergy of esketamine and propofol reduces PONV as well as other short-term adverse events, thereby providing a better safety and satisfaction profile of ERAS for laparoscopic appendectomy and cholecystectomy.

## Trial status

The first patient was included on October 1, 2021. We expect to finalize the study in September 2022.

## Data Availability

Not applicable
